# PFOA and testis cancer in the Veneto Region (Italy)

**DOI:** 10.1186/s12940-024-01064-4

**Published:** 2024-03-28

**Authors:** Mario Saugo, Enrico Ioverno, Armando Olivieri, Francesco Bertola, Angela Pasinato, Alan Ducatman

**Affiliations:** 1 College of Physicians of the Province of Vicenza, Vicenza, Italy; 2 West Virginia University, Morgantown, WV US

**Keywords:** PFOA (perfluorooctanoic acid), PFAS (per- and polyfluoroalkyl substances), Testicular Cancer, Public Health Surveillance

## Abstract

The largest documented episode of human contamination by PFOA in the world (approximately 150,000 actual residents on 1 January 2020) has occurred in Italy’s Veneto Region. In this large, mostly flat plain area, a cluster of testicular cancers has also been observed. Preliminary data are reported, and the most relevant and recent recommendations regarding the health surveillance of exposed individuals are emphasized.

## Background

The International Agency for Research on Cancer (IARC) has recently drawn public attention to PFOA (Class I) and PFOS (Class IIb) as carcinogens, with the strongest evidence in humans for kidney cancer followed by testicular cancer. Among new evidence, the IARC Working Group took into consideration an ecological analysis of rates of orchiectomy, an indicator of testicular cancer, conducted in the Veneto Region (Italy) [1]. English-speaking readers may appreciate some additional details, as the original report is in Italian [2].

## Testicular cancer evaluations in the Veneto Region

Extensively documented human exposures to high dose PFOA are from communities impacted by industrial pollution, such as the well-known case in the mid-Ohio Valley, United States, in the Veneto Region, Italy, while another relevant episode of PFAS drinking water contamination featured a firefighting foam source, and exposure to perfluorooctanesulfonic acid (PFOS) and perfluorohexane sulfonic acid (PFHxS) in Ronneby, Sweden [3–5]. Appreciation for the contamination in the Veneto Region emerged in 2013 and represents the widest episode of PFOA human exposure described worldwide.

At the time the above mentioned Venetian report was drafted in 2016 [2], the available data from the Veneto Region Cancer Registry concerning the impacted area covered only the period 2010–2013 [6]. Case-finding was therefore extended, abstracting orchiectomies for testicular cancer in males aged 15–54 in the Veneto Region from 1997 to 2014 from the archives of Hospital Discharges (ICD-9-CM 186 code for diagnosis and 62.3–62.4 codes for surgical procedures). When considering data from a non-impacted area of the Veneto Region with IARC-certified cancer registration, this approach showed high sensitivity (92%, 95% CI 87%-95%) and positive predictive value (90%, 95%CI 85-93%) for the diagnosis.

Each case was assigned the date of admission and the municipality of residence recorded at hospital admission. Expected cases were calculated using indirect internal standardization: five-year age group-specific cumulative incidence rates in the Veneto Region were applied to each quinquennial strata of the average population residing in each of the 21 municipalities that constituted the most impacted area. The affected region was divided into subareas A and B. In subarea A, drinking water supplies and groundwater were contaminated, resulting in heavier contamination of private wells. In subarea B, delivered drinking water supplies were contaminated, but the groundwater remained unaffected.

Of note, no excess cases were observed for the entire area (Standardized Incidence Ratio – SIR: 1.02, CI 95% 0.81–1.29) or subareas A (SIR: 1.19 CI 95% 0.89–1.58) and B (SIR: 0.80 CI 95% 0.53–1.20), while the municipality of Lonigo (the largest one located in subarea A) showed 16 observed testicular cancer cases compared to 8.7 expected cases (SIR 1.84, 95% CI 1.05–2.98) [2]. This municipality also exhibited very high and long-lasting exposure to PFOA contaminated drinking water from public water supplies or private wells, and very high PFOA serum concentrations among residents, and resident farmers [7, 8]. The observation is potentially important because of previous findings from the large (*n* = 69,030) enrolled population of the C8 Health Project in the Mid-Ohio Valley of the United States. In that population the excess of testicular cancer cases concentrated in the heavily PFOA-exposed Little Hocking water district, and among those with serum PFOA concentrations above 110 ng/mL and at least 10 years of exposure [9].

An ecological mortality study also suggested a link between testicular cancer mortality and PFOA exposure in the contaminated area of the Veneto Region in 1980–2013, with 8 observed vs. 4.3 expected cases [10]. A recent Age-Period-Cohort ecological mortality study, still undergoing its peer-review, restricts the analysis to period 1985–1999 (5 observed vs. 1.95 expected deaths, for a SMR of 256 (90% CI: 101–539; one-sided mid-p value = 0.032) [11]. Mortality from testicular cancer has greatly improved due to advancements in oncological treatments), and the information from a mortality study will address a minority of cases.

The present report concerning the geographic sub distribution of testicular cancer cases in Veneto is preliminary, and additional follow-up of the impacted population of the Veneto Region with refined ecological and analytical research for incident cases is needed.

While research is ongoing, the residents of contaminated areas in Veneto have reasons to request opportunities to safeguard their health. Since November 2013, residents of affected communities in the mid-Ohio Valley of the United States have been eligible for health surveillance, including medical surveillance for testicular cancer and kidney cancer [12]. In the United States, recommendations for PFAS health surveillance have also been made in 2022 by an expert committee empaneled by the US National Academies of Sciences, Engineering, and Medicine [13]; and by the PFAS-REACH program in 2023. The PFAS REACH program, funded by the US National Institute of Environmental Health Sciences, and led by Silent Spring Institute in collaboration with universities and community partners, has succinct recommendations for patients and clinicians in affected communities including testicular cancer surveillance [14].

For testicular cancer, US recommendations for screening have included medical history and physical examination, including testicular inspection, with additional testicular ultrasound if needed; for renal cancer among adults: medical history, physical examination, and for red blood cells in the urinary sediment, with additional renal ultrasound examination if needed [12–14].

The College of Physicians of the Province of Vicenza has drawn the attention of its members to this issue.

## Data Availability

No datasets were generated or analysed during the current study.
